# Supplementary figures and images for: Sample-level sound synthesis with recurrent neural networks and conceptors
Source: PeerJ Comput Sci. 2019 Jul 8;5:e205. doi: 10.7717/peerj-cs.205 (PMC7924416; doi:10.7717/peerj-cs.205)

An 11 point morph between a snare and bongo sound, created by mixing conceptors

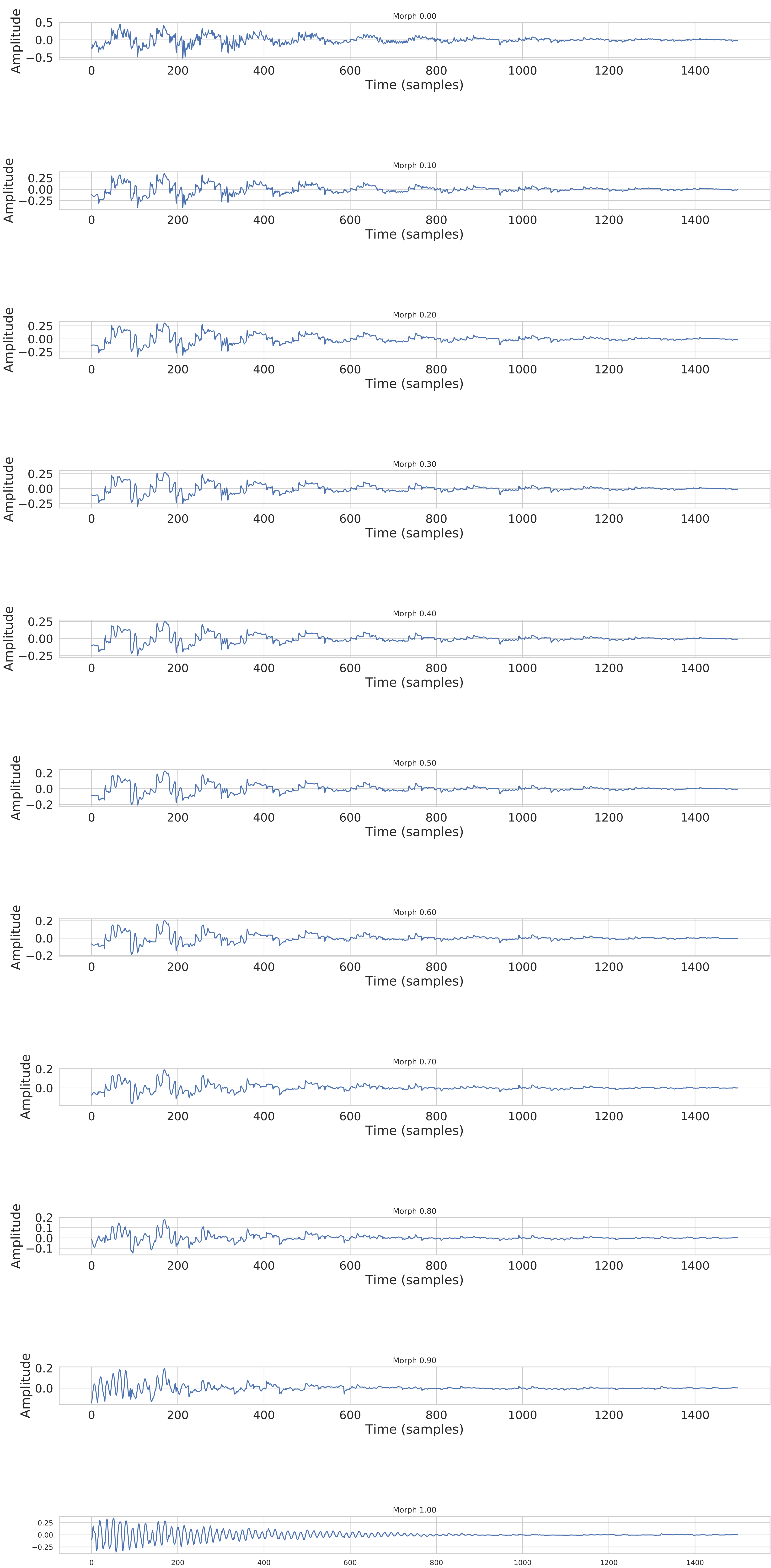

Supplement: Figure S1 — The waveforms represent each stage of an 11-point morphing process. [file peerj-cs-05-205-s013.pdf]

An 11 point linear mix between a snare and bongo sounds

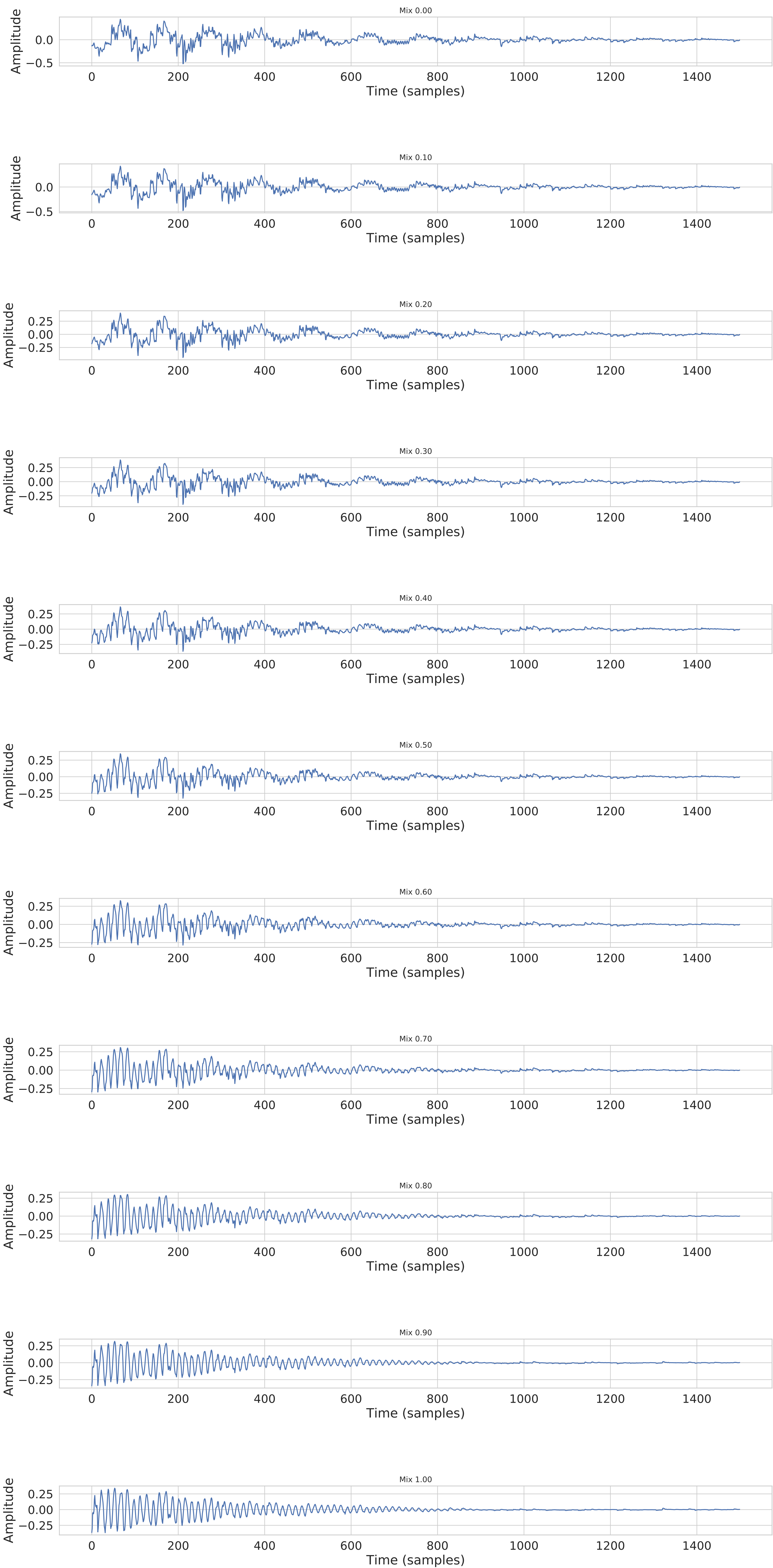

Supplement: Figure S2 — The waveforms represent each stage of an 11-point mix of amplitude values. [file peerj-cs-05-205-s014.pdf]
